# Post-mortem persistence of SARS-CoV-2: a preliminary study

**DOI:** 10.1007/s12024-021-00375-z

**Published:** 2021-05-08

**Authors:** Sara Sablone, Biagio Solarino, Davide Ferorelli, Marcello Benevento, Maria Chironna, Daniela Loconsole, Anna Sallustio, Alessandro Dell’Erba, Francesco Introna

**Affiliations:** 1 Section of Legal Medicine, Department of Interdisciplinary Medicine, Bari Policlinico Hospital, University of Bari, 70124 Bari, Italy; 2 Section of Hygiene, Department of Biomedical Sciences and Human Oncology, Bari Policlinico Hospital, University of Bari, 70124 Bari, Italy

**Keywords:** SARS-CoV-2, Viral RNA, Cadaver tissue samples, Post-mortem interval, COVID-19 autopsy

## Abstract

Since the beginning of March 2020, the severe acute respiratory syndrome coronavirus 2 (SARS-CoV-2) pandemic has been the cause of millions of deaths worldwide. The need to better define the pathogenesis of coronavirus disease 19 (Covid-19) as well as to provide the correct statistical records concerning deaths related to this virus, inevitably involves the role of forensic pathology and routine autopsy practice. Currently, some data on macroscopic and microscopic features in autopsies performed in suspected Covid-19 cases are reported in the literature. The persistence of SARS-CoV-2 in cadavers has not yet been elucidated and only a few reports have emphasized the importance of evaluating the Virus RNA in post-mortem tissues. In this preliminary study, we observed that SARS-CoV-2 survives in multiple cadaver tissues many days after death despite some extreme conditions of post-mortem body preservation. The results of this on-going analysis could help improve the safety of working practices for pathologists as well as understanding the possible interaction between microbiological agents and the cadaver tissue’s supravital reactions.

## Introduction

SARS-CoV-2 has caused millions of deaths worldwide. Globally, new COVID-19 cases and deaths have continued to increase to approximately 4.6 million new cases and about 79 000 new deaths every week. This brings the cumulative number to over 75 million reported cases and 1.6 million deaths globally since the start of the pandemic [1].

In the living, the virus spreads through respiratory droplets and the conjunctival and bronchial epithelium are mentioned as portals for infection [2]. SARS CoV-2 Ribonucleic Acid (RNA) detection on respiratory specimens is considered the gold standard for diagnosing the infection. Bilateral pneumonia progressing to acute respiratory distress syndrome (ARDS) represents the most often reported SARS-CoV-2 related cause of death. Against this backdrop, autopsy practice has a fundamental role in better defining the pathogenesis of SARS-CoV-2 related disease and providing accurate epidemiological data upon confirmed Covid-19 death cases [3].

From a forensic pathology standpoint, only a few articles have focused on macroscopic features and microscopic findings in those who have died from SARS-CoV-2 infections.

The virus risk of transmission is alleged even in autopsy routine practice, and international societies proposed to classify SARS-CoV-2 in risk group 3 [4].

Currently, no virus infections in forensic pathologists or medical staff involved in mortuary or autopsy practice have been reported in the literature. This means that if appropriate protective measures are followed, an increased risk of transmission is not expected in these professions [5].

Though the persistence of human coronaviruses on inanimate surfaces has been seen for up to nine days [6], the evidence of SARS-CoV-2 in cadavers is still a matter under discussion. Beltempo and colleagues reported the persistence of SARS-CoV-2 RNA in respiratory swabs taken 35 days post-mortem. Therefore, a question arises about the detectability of the virus under particular conditions of post-mortem body preservation [7].

With this in mind, we detected SARS-COV-2 RNA on samples taken on whole tissues of cadavers buried and/or frozen at minus 15 °C for many days before the autopsy. All the possible implications of these results will be discussed here.

## Materials and methods

From October to December 2020, 5 judicial autopsies were performed at the Legal Medicine Unit of the Bari Hospital. Four of the cadavers were male and one was female. The ages ranged from 44 to 84 years (Mean: 63.8). The cause of death for 4 decedents was "Coronavirus 19-related bilateral pneumonia producing acute respiratory failure" (ICD-10-CM: J12.82). In one case, it was "Polytrauma from suicidal precipitation in Covid affected patient" (ICD-10-CM: X80).

In two cases, death occurred in the Emergency Department, in one case in the Intensive Care Unit, in another case at the Infectious Disease Unit, and another at home.

The time interval between the moment of death and the autopsy varied between 22 and 27 days. During this interval, the bodies underwent a period (from 20 to 25 days) in a refrigerator at a temperature of minus 15 °C. The bodies were allowed to reach room temperature before the autopsy. One cadaver was also buried for 5 days before being placed in the refrigerator. The main features are summarized in Table 1.

**Table 1:** Cadaver’s principal features

Case	Age	Sex	Cause of death	Place of death	Interval death—autopsy	Interval T°C (Days)
1	59	M	SARS-CoV-2 related bilateral pneumonia producing acute respiratory failure	Intensive Care Unit	22,5 days	21 (-15 °C); 1,5 (Room Temperature)
2	84	M	Polytrauma from suicidal precipitation in Covid-affected patient	Home	24,5 days	23 (-15 °C); 1,5 (Room Temperature)
3	68	M	SARS-CoV-2 related bilateral pneumonia producing acute respiratory failure	Emergency Department	23,5 days	22 (-15 °C); 1,5 (Room Temperature)
4	44	F	SARS-CoV-2 related bilateral pneumonia producing acute respiratory failure	Emergency Department	26,5 days	5 (Buried); 20 (-15 °C); 1,5 (Room Temperature)
5	64	M	SARS-CoV-2 related bilateral pneumonia producing acute respiratory failure	Emergency Department	22,5 days	21 (-15 °C); 1,5 (Room Temperature)

Before an anatomical examination, a sample extraction plan was made by consulting clinical pathologists, laboratory staff, and virologists. To standardize and record the sampling procedures, an autopsy checklist was prepared. Samples were taken directly after opening body cavities; organ and tissue cuttings were minimized (compatibly with investigative needs) to reduce infection risks. Post-mortem multifocal swabs were performed to gain samples of body fluids, while tissue blocks were collected and stored in 2 ml-plastic test tubes (Eppendorf). As soon as the autopsies were over, all cadaveric samples were sent to the Laboratory of Molecular Epidemiology and Public Health of Hygiene Unit, Bari Hospital, to undergo molecular tests.

Molecular detection of viral RNA in cadaveric specimens was performed by extracting RNA from 5 μg of tissue biopsy, preliminary overnight incubation in a lysis buffer RLT, and UTM (Copan, USA) swabs. Extraction was performed using the Nimbus platform (Hamilton, USA), and then PCR was performed using the Allplex™ 2019-nCoV assay (Seegene, South Korea) on the CFX96 (Bio-Rad, USA) in line with the manufacturer's instructions. The gene targets for the PCR assay were the N gene, the E gene, and the RdRP gene. An internal control provided assurance that specimens were successfully amplified and detected. The cycle threshold (Ct) value was recorded for each of the 3 genes. Samples with a detected result for all 3 genes, or a single target gene, were interpreted as SARS Cov2 PCR positive, in line with the manufacturer's guidance.

Researchers in clinical studies have not validated the use of Ct to guide the management of COVID-19 cases. Indeed, Ct values from viral RNA can vary depending on the method of specimen collection, specimen source, transport, and the time from infection to collection to analysis. Additionally, no standard exists to validate quantitative assays that produce comparable results from labs and manufacturers.

Samples showing the amplification of the internal control but not the detection of the gene targets were classified as negative. Samples not showing the amplification of internal control were classified as invalid.

## Results

Some of the tested samples did not produce results due to the presence of inhibitory factors and we have indicated these with the acronym N/A (Not Assessed). The remaining samples provided results, which we expressed in terms of Ct (Cycle threshold). Ct refers to the number of cycles needed to amplify viral RNA to reach a detectable level. A high CT value might not be considered for accurate identification of the infectious potential of a patient [8–10].

We used Ct to indicate the positive outcome of the molecular tests on most of the analyzed samples, suggesting a multi-organ dissemination of Coronavirus-2.

**Fig. 1:**
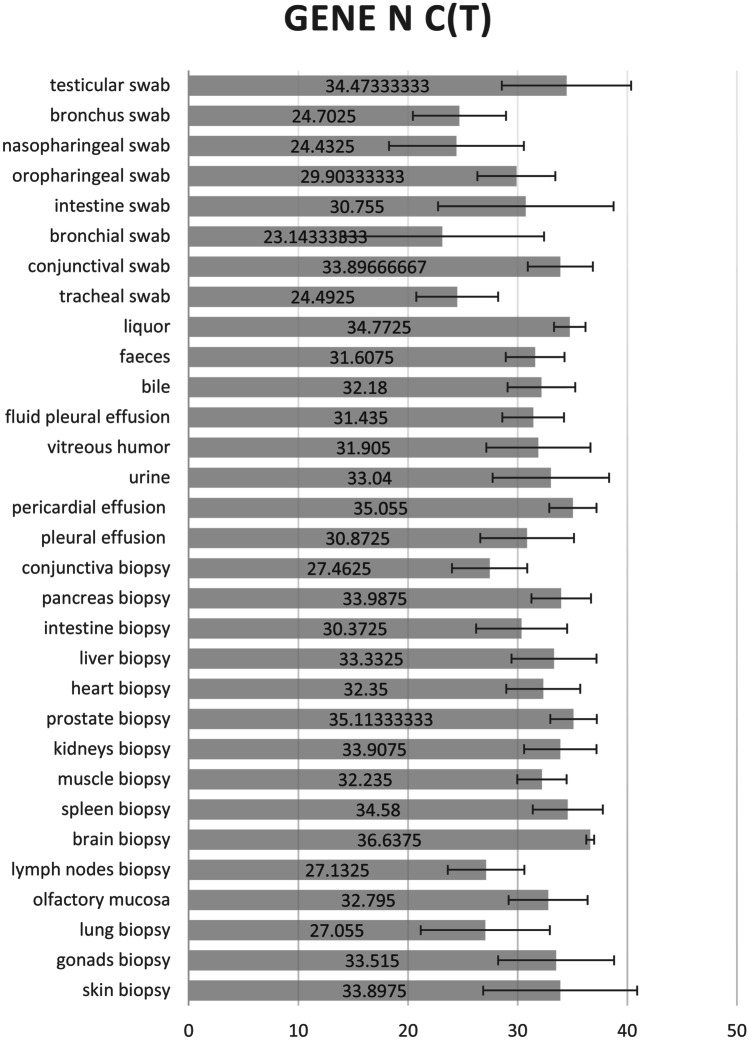
Averages of the N Gene Ct values obtained for each tissue sample analyzed

**Fig. 2:**
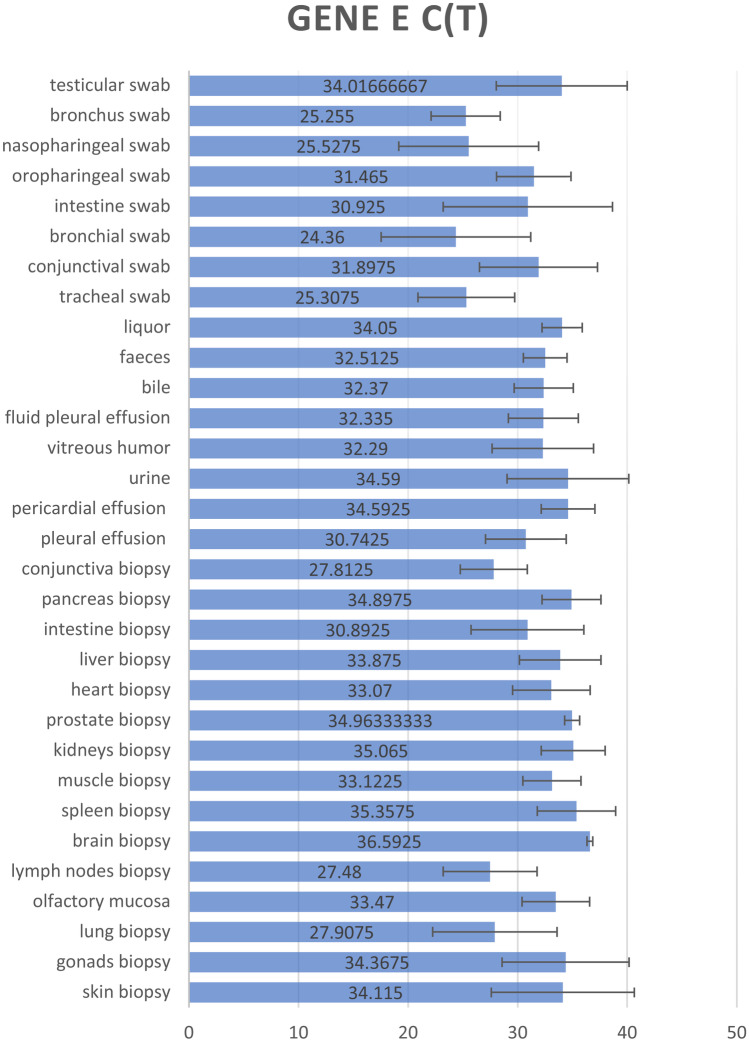
Averages of the E Gene Ct values obtained for each tissue sample analyzed

**Fig. 3:**
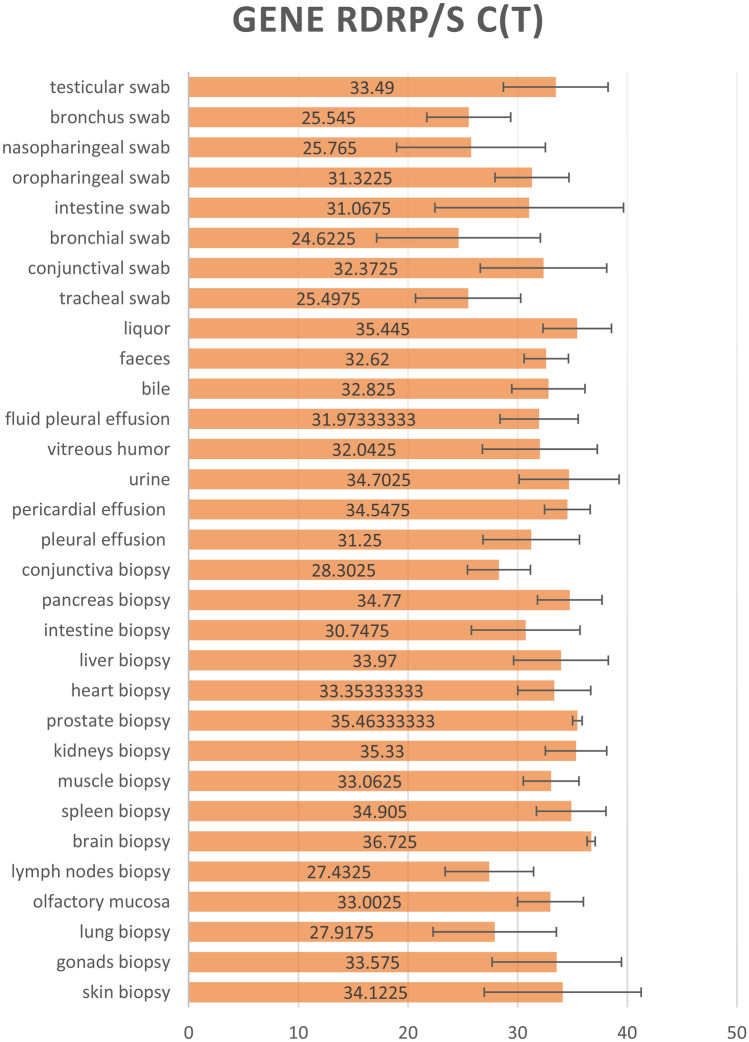
Averages of the RdRP Gene Ct values obtained for each tissue sample analyzed

We report the averages of the CT values obtained for each tissue sample analyzed in graphs (Figs. 1, 2, and 3).

Time from death to post-mortem examination ranged from 22 to 27 days. In one case, the body had also been buried for 5 days. Swabs were positive for SARS-CoV-2 by RT-PCR in all cases regardless of the timing of post-mortem examination and even though the bodies had stayed for several days in a cold room at -15 °C and one of them had previously been buried for 5 days.

## Discussion

Coronavirus disease 2019 (COVID-19) is regarded as number three in the line of extremely infectious human coronavirus (such as the Severe Acute Respiratory Syndrome [SARS] and Middle East Respiratory Syndrome [MERS] coronaviruses) that have appeared over the last 20 years [11–13]. The unfolding of the novel COVID-19 pandemic has resulted in a significant burden on the global medical community, including autopsy activity [14, 15].

According to the World Health Organization (WHO), the transmission of severe acute respiratory syndrome coronavirus 2 (SARS-CoV-2) between individuals is through inhalation of sizeable respiratory droplets, fomites, and proximity with infected persons and facets [16]. Although, as at the time the WHO released its report in March 2020, there was no proof of people being infected as a result of exposure to COVID-19 related dead bodies [17], the suspected risk to health professionals of COVID-19 infection from an individual whose death resulted from COVID-19 could not be overlooked.

For this reason, the Italian Ministry of Health issued a recommendation that in principle discourages the performing of autopsies on COVID-19 related bodies [18]. However, the autopsy is of great significance for elucidating the pathological changes, pathogenesis, and cause of death of Coronavirus Disease 2019 (COVID-19) and can provide a theoretical basis for prevention and control of the outbreak [19, 20]. Moreover, the more we investigate SARS-CoV-2 ability to survive in different environments, the safer autopsies on individuals with COVID-19-related death will be.

According to the relevant literature, the majority of the microorganisms responsible for death also have a shorter life-span after the death of the host and therefore infection risk is minimized [21]. The higher risk that pathologists would be infected during the autopsy is generally attributed to common viral agents (HIV, HBV, HCV), mycobacterium tuberculosis, and the prions responsible for Transmissible Spongiphorme Encephalopathis [22]. Such pathogens are included in the infection Hazards Groups 3, where the risk to mortuary staff is minimal if standard safety autopsy procedures are applied [23].

Nonetheless, there is the possibility of an increase in the chances of disease transmission with direct exposure to body fluids and invasive procedures on the cadaver, such as procedures that generate droplets or aerosols [17, 24]. Soft tissues like the lungs of the cadaver could also be infectious if not handled properly during an autopsy [25]. Moreover, albeit less frequently, it is postulated that transmission may occur via the contamination of inanimate surfaces from an activated virus.

The SARS-CoV-2 virus has strong persistence in the external environment, especially at low temperatures. Kampf et al. supported the claim that human coronaviruses on inanimate surfaces could still be contagious at room temperature for about nine days [26]. However, this duration of persistence could be reduced at a temperature of 30 °C or higher. The WHO confirmed that human severe acute respiratory syndrome coronavirus 2 (SARS-CoV-2) could still be contagious on surfaces for as long as nine days [24].

According to a study on the resistance of severe acute respiratory syndrome coronavirus [27], the virus can survive for 2 days in hospitals or domestic sewage and dechlorinated tap water, for 3 days in feces, 14 days in normal saline, and 17 days in urine at 20 °C away from light. However, at 4 °C, the virus can survive longer than 14 days in the abovementioned water settings, and longer than 17 days in feces.

In a study conducted by Riddell et al. survival rates of SARS-CoV-2 were determined at different temperatures on common surfaces [28]. The authors obtained half-lives of between 1.7 and 2.7 days at 20 °C, reducing to a few hours when the temperature was elevated to 40 °C. With initial viral loads broadly equivalent to the highest titres excreted by infectious patients, the viable virus was isolated for up to 28 days at 20 °C from common surfaces such as glass, stainless steel, and both paper and polymer banknotes. Conversely, infectious viruses survived less than 24 h at 40 °C on some surfaces.

Pagat et al. argued that heat (autoclave) and hypochlorites are effective in the decontamination of SARS-CoV [29], while other studies demonstrate that refrigeration may promote survival and persistence of coronaviruses [24, 30]. It is also currently accepted that formalin fixation at room temperature inactivates SARS-CoV-2 within 24 h [31]. Moreover, the processes of paraffin embedding that occur at high temperature (60–65 °C) seems to contribute to virus inactivation [32].

In human inanimate bodies, a report by Camero stated that people who had passed away from the novel COVID-19 might still be contagious for hours or days [33]. In a series of 79 bodies examined by Heinrich et al. virus isolation by nasopharyngeal swabs was demonstrated up to 35 h post-mortem [34]. In a case report by Beltempo et al., the persistence of SARS-CoV-2 RNA in the upper respiratory tract of a patient dying with COVID-19 was documented 35-days after death [7].

In consideration, therefore, of the potential relevance of data generated by analysis of material obtained from post-mortem examination, adopting the stricter safety procedures, we performed 5 complete autopsies with the main objective of verifying SARS-CoV-2 persistence in individuals whose death resulted from COVID-19 and whose cadavers were refrigerated at a temperature of -15° for a time interval between the moment of death and the autopsy which varied between 22 and 27 days. One of them had previously been buried for 5 days.

Our study demonstrates that a large amount of virus, which has a multiorgan distribution thanks to its tropism for many human organs and tissues, may remain in deceased people infected with SARS-CoV-2, and the survival time of the virus may even be prolonged in refrigerated cadavers thanks to its resistance to cold environments.

Thus, the performance of autopsies on infectious COVID-19 bodies could be catastrophic for forensic pathologists and mortuary workers if the cadavers are not properly handled. As a rule in the COVID-19 pandemic, some invasive procedures in standard pathology autopsies, especially those methods that produce tiny-particles (such as using an oscillating saw or cleansing of intestines) should be discouraged. However, if it is necessary to perform any of the above procedures, full protection with necessary personal protective equipment (PPE) is vital [18, 35, 36].

In the light of the current COVID-19 pandemic, it is pertinent to ensure that COVID-19 safety protocols and standard operating procedures recommended for cadaveric dissections are strictly followed [37–40]. Besides, personal protective equipment (PPE) such as an impermeable disposable gown or overall, hand gloves, and face protectors (medical mask, shield, and goggles) should be provided for mortuary staff and other personnel that handle both the embalmed bodies and cadaver dissection [17, 25, 41].

## Conclusions

The results of our study demonstrate that personal protection of examiners and disinfection of dissecting rooms, surroundings, and instruments should be taken seriously to avoid examiners being infected due to inadequate protection or careless operation.

At the same time, as the remains of patients who have died of COVID-19 may have a large amount of the virus, the prevention of infected bodies being accessible to relatives and speeding up the post-mortal procedures for burial is thoroughly justified.

Our study demonstrates that SARS-CoV-2 infection is characterized by a multiorgan distribution as this coronavirus has a tropism for many human organs and tissues. Nevertheless, such multiorgan distribution in cadaver tissues does not mean that infected people had developed the disease during their lifetime, nor is it related to disease severity. For this reason, matching molecular investigation results with any detectable anatomical damage by histological analyses of infected tissues is essential, and this is the subject of an ongoing study by the authors. Certainly, the ubiquitous post-mortem presence of SARS-CoV-2 even a long time after death and also in bodies subjected to burial and/or refrigeration is an extraordinary result, demonstrating the high resistance of this coronavirus to uncomfortable environmental conditions.

However, these results must be treated with caution, not only because positive molecular investigations for coronaviruses are not automatically diagnostic of coronavirus disease, but also and above all because they do not always define the main cause of death. Therefore, without contextual histological investigations or when the results of histological investigations are compromised by partial cadaveric decomposition or by advanced putrefaction, the positivity of post-mortem molecular analyses should not lead pathologists to conclude that COVID-19 was the cause of death, especially due to the consequences that such improper conclusions could have in medico-legal disputes.

## Key points

1. The SARS-CoV-2 virus has strong persistence in the external environment, especially at low temperatures, and it could still be contagious on inanimate surfaces.
2. A large amount of virus may remain in deceased people infected with SARS-CoV-2 even a long time after death and also in bodies subjected to burial and/or refrigeration. The survival time of the virus may even be prolonged in refrigerated cadavers thanks to its resistance to cold environments.
3. The ubiquitous post-mortem presence of SARS-CoV-2 RNA demonstrates that SARS-CoV-2 infection is characterized by a multiorgan distribution, thanks to virus tropism for many human organs and tissues.
4. The suspected risk to health professionals of COVID-19 infection from an individual whose death resulted from COVID-19 should not be overlooked.
